# New Generation of Antibacterial Products Based on Colloidal Silver

**DOI:** 10.3390/ma13071578

**Published:** 2020-03-29

**Authors:** Bogdan Pascu, Adina Negrea, Mihaela Ciopec, Corneliu Mircea Davidescu, Petru Negrea, Vasile Gherman, Narcis Duteanu

**Affiliations:** Faculty of Industrial Chemistry and Environmental Engineering, Politehnica University of Timisoara, 2 Piata Victoriei, RO 300006 Timisoara, Romania; ioan.pascu@upt.ro (B.P.); corneliu.davidescu@upt.ro (C.M.D.); petru.negrea@upt.ro (P.N.); vasile.gherman@upt.ro (V.G.); narcis.duteanu@upt.ro (N.D.)

**Keywords:** colloidal silver, adsorption, band aid, ultrasonic, soluble starch, antibacterial effect

## Abstract

The main objective of the present paper is the green synthesis of colloidal silver by ultrasonication starting from silver nitrate and using soluble starch as the reducing agent. Soluble starch has been used during synthesis because it is a cheap and environmentally friendly reactive. Silver colloid has been characterized by physicochemical methods: UV–VIS spectroscopy, Scanning Electron Microscopy and Energy Dispersive X-Ray spectroscopy. This colloidal material was prepared in order to prove and establish its toxicity on heterotrophic bacteria. Toxicity tests were carried out using test cultures with and without silver colloid with different concentrations. This way was possible to establish the minimum silver concentration that presents a toxic effect against used bacteria. Quantitative evaluation of bacterial growth was performed by using the Most Probable Number method. By counting the bacterial colony number, the antibacterial effect was determined for colloidal silver deposited onto the cotton gauze by adsorption. During the present study, we optimized the adsorption specific parameters: solid:liquid ratio, temperature, contact time, colloidal silver concentration. By thermodynamic, equilibrium and kinetic studies, the adsorptive process mechanism was established.

## 1. Introduction

Technological development from the last decade lead to extensive usage of colloidal silver in different fields increasing the number of possible applications. Due to the mechanical and physical-chemical properties, colloidal silver is used in several fields: electronics, electrotechniques, biotechnology, bioenergy, textile and optical industries, pharmacy, medicine and environmental protection [1,2]. An extensive literature review proved that colloidal silver has a large number of applications: antibacterial agent [3,4], antifungal agent [5], antiviral agent [6], air disinfection [7], water disinfection [3], ground water biological wastewater treatment [8] and surface disinfection (antimicrobial paints, plastic catheters, gel formulation for specific usage, packing paper for food preservation, clinical clothing) [3].

**Figure 1:**
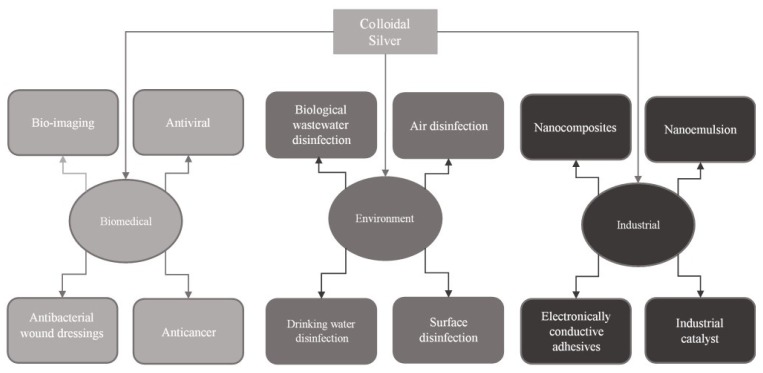
Silver colloidal applications.

Schematically, colloidal silver applications are presented in Figure 1.

A versatile material with multiple uses in textile and medical fields is cotton because it is a soft material and has good adsorbent properties [2,9,10,11]. A big problem for usage of textile materials in the medical field is represented by the fact that all textile materials are exposed to several viruses and bacteria [12]. Protective level of gauze is dependent on external layer hydrophobicity; such layer being called the protective layer. If the protective layer is not resistive to a microorganism’s presence, it may pose a risk for the health of the patients. Different microorganisms can grow exponentially if the temperature and humidity are optimal. Starting from this point, it is possible to reduce and minimize the infections with different pathogen agents by using different textile materials with improved antimicrobial properties [13,14,15,16,17,18]. Experimental data proved that some metals, such as silver or gold, or some metallic oxides (titanium oxide, zinc oxide or cooper oxide) present good antimicrobial activity [2,16,17,18,19,20,21,22,23,24,25,26,27,28,29].

By correlating this observation with the necessity for different textile materials with good antimicrobial properties, we aim to prepare a new type of medical bandage by modifying a cotton bandage with an antimicrobial layer of colloidal silver. In the newly prepared medical bandages, an improved antimicrobial property silver layer was introduced by adsorption.

Colloidal silver can be prepared using physical methods (evaporation, condensation) [30,31], or chemical methods (microwave synthesis, microemulsion techniques, chemical reduction) [32,33,34,35], photochemical methods [36,37,38], biological methods using some microorganisms (*Pseudomonas stutzeri*) [39,40] or different plant extracts: *Garcinia mangostana* [35], *Cinnamomum tamala* [41].

Such colloidal material can be obtained only if the colloid stabilization is realized rapidly after silver reduction. Reduction process can be realized by using different reducing agents: sodium citrate, ascorbate [42], sodium borohydride [43], elemental hydrogen, different polyols, N,N dimethyl formamide (DMF) [32], ascorbic acid, poly (ethylene glycol)–block copolymers [44], hydrazine, ammonium formate [34].

In the present paper, soluble starch has been used as the reducing agent because it is a green reactant and due to its low price. Reduction process was conducted using ultrasonication in order to assure and maintain proper contact between reagents, due to the propagation of ultrasonic waves in liquid media. In this propagation process, it was observed that compression cycles alternate with lower pressure cycles when a liquid rarefication is observed leading at cavitation process [45]. During the compression cycle, high temperature is created in the liquid. In addition, it was observed that during cavitation the contact surface between solid support and liquid molecules increases. Cavitation phenomenon occurred simultaneously in several points inside of the liquid, leading to high temperatures and pressures which are responsible for further physical-chemical modifications [46,47].

The aim of the present paper was to prepare a medical bandage with antimicrobial properties by deposition of colloidal silver on cotton gauze. After preparation of such advanced medical bandages, antimicrobial activity has been tested. These tests were carried out by studying the behavior of environmental heterotroph microorganisms when they are exposed to different colloidal silver concentrations. Heterotroph microorganisms were used because they have a good resistance to different drugs, different structures and different metabolism.

## 2. Materials and Methods

### 2.1. Biocide Efect of Colloidal Silver Solution

Long time usage of silver particles proved that they present a good biocide effect against a large spectrum of Gram-positive and Gram-negative bacteria. Starting from this observation, our aim was to test the biocide effect of prepared colloidal silver. During the experiments we used colloidal solutions with silver concentrations between 1.2 μg L^−1^ and 116 mg L^−1^.

The material designed by us is a new material, therefore it is preferable for the toxicological tests to be carried out using bacterial inoculations that come from natural environments with a broader spectrum of Gram-positive and Gram-negative bacteria. Thus, an inoculum of water from the Bega River has been used, which contains a wide spectrum of Gram-positive and Gram-negative bacterial species. These bacteria were grown using a solid non-selective growth medium plate count agar—Himedia (Enzymatic Digest of Casein 5.0 g/L; Yeast Extract 2.5 g/L; Dextrose (Glucose) 1.0 g/L; Agar 15.0 g/L; Final pH: 7.0 ± 0.2 at 298.15 K), preparing two different types of cultures: test cultures were prepared on plates with different colloidal silver content, and control cultures prepared on plates without colloidal silver content.

Bacteria cultures were prepared in two different ways, for control cultures (3 repeats) in Petri dishes containing 10 mL plate count agar growth medium were introduced 1 mL of bacterial inoculum and 1 mL distilled water. For test cultures (3 repeats) in Petri dishes containing 10 mL plate count agar were introduced 1 mL bacterial inoculum, and 1 mL from colloidal solution. All used Petri dishes were incubated at 303 K for 48 hours. After incubation the quantitative bacterial growth was estimated using the Most Probable Number method (counting the bacterial colonies) by comparing it to the bacterial control culture.

### 2.2. Antibacterial Activities

Studies of antibacterial properties of colloidal silver were conducted by using test medical bandages on which surface colloidal silver had been deposited by using a thermostatic bath. The influence of different parameters (solid material: liquid ratio, silver initial concentration and temperature) were evaluated on the adsorptive capacity of medical bandage used during tests. Optimum ratio solid:liquid was established by varying the number of gauze pieces (with dimension of 20 × 20 mm) between 5 and 70 for the same volume of colloidal silver (25 mL) with a concentration of 50 mg L^−1^. All adsorption experiments were carried out at pH 4 and 273 K. Time contact and temperature influences on adsorption capacity were studied by varying the contact time between 1 and 6 hours and the temperature between 298 and 318 K for the optimum solid:liquid ratio = 30 pieces of gauze: 25 mL colloidal silver at pH 4. 

Equilibrium concentration was established by using different initial concentration of colloidal silver (5, 10, 15, 25, 40, 50, 60 and 75 mg L^−1^) for the same solid:liquid ratio = 30 pieces of gauze: 25 mL colloidal silver. All adsorptions were carried out at 298 K, pH = 4 for 4 hours.

From all batch experiments medical bandages were extracted from solutions and in the obtained solutions we determined silver residual concentration by atomic absorption spectroscopy (Varian SpectrAA 280 FS). Obtained pieces of medical bandages were dried at 303 K for a minimum of 24 h and tested for antimicrobial effect for further usage in the medical field.

Antibacterial tests on the prepared medical bandages were performed on Bega river heterotrophic bacteria cultures (Gram positive and Gram negative bacteria). All bacterial cultures were obtained on a solid non-selective growth medium (plate count agar), making two types of cultures: test cultures (3 repeats) using silver impregnated medical bandages, and control cultures in which were used un-impregnated medical bandages with the same shape and size like the samples used in test cultures. Medical bandage pieces were placed into the Petri dishes at a distance of 2 cm from dish walls, after that was added the plate count agar medium and the entire system was inoculated with 600 μL of Bega water, with content of heterotrophic bacteria. The obtained system was incubated at 303 K for 48 h. After incubation we evaluated the growth of the bacteria for test cultures and for control cultures.

### 2.3. Synthesis of Colloidal Silver 

#### 2.3.1. Effect of Precursor: Reducer Ratio 

Colloidal silver synthesis was carried out by using soluble starch (analytical purity, Merck) as the reducing agent. An important factor during synthesis is represented by the ratio between silver precursor, reducing agent and water. As a precursor for colloidal silver preparation, silver nitrate was used, analytic grade from Merck. During synthesis, different quantities of reducing agent were used: 0.5, 1, 2 and 4 g, respectively, for the same quantities of silver precursor (4 g) and solvent volume has been kept constant for all experiments (20 mL of distilled water). By using these quantities of reagents we obtained ratios: 0.25:2; 0.5:2; 1:2; 2:2. During preparation, all samples were mixed for a minimum of 60 minutes at 353 K using an ultrasonic bath with a power of 320 W.

#### 2.3.2. Effect of Ultrasonication Time

Through preliminary attempts were conducted experiments for sonication time up to 240 minutes, but the obtained results were similar with the results obtained for 90 minutes. Based on that, in the present research paper the sonication time was varied between 30 and 90 minutes. Effect of ultrasonication time was studied by varying the mixing time from 30 to 90 minutes at 353 K for a ratio of silver precursor:reducing agent = 1:2.

#### 2.3.3. Effect of Ultrasonication Temperature

Temperature influence has been studied by varying the temperature between 333 and 353 K, for an ultrasonication time of 60 minutes using a ratio of silver precursor:reducing agent:water = 1:2:10.

### 2.4. Characterization of Colloidal Silver Materials

Presence of silver particles into the prepared silver colloid was proved by recording the UV–VIS spectra using Varian Cary 50 spectrophotometer, and by recording the scanning electron micrographs using a Quanta FEG – 250 Scanning Electron Microscope.

### 2.5. Kinetics and Thermodynamic Studies

Kinetics of the colloidal silver adsorption process on medical bandages have been studied by modeling obtained experimental data with two kinetic models: pseudo-first-order model [48] and pseudo-second-order one [49,50].

It is well known that the activation energy represents the minimum kinetic energy of reactants in order to react. Activation energy can be evaluated from Arrhenius equation:

(1) $$\ln k_{2}=\ln A-\frac{E_{a}}{RT}$$

where: *k_2_*—speed constant (g min^−1^ mg^−1^), *A*—Arrhenius constant (g min mg^−1^), *E_a_*—activation energy (kJ mol^−1^), *T*—absolute temperature (K), R—ideal gas constant (8314 J mol^−1^ K^−1^).

Adsorption processes can be characterized and optimized by evaluating the free Gibbs energy, enthalpy and entropy. Values of thermodynamic function Gibbs free energy (ΔG^0^) can be calculated by using the following relation:

(2) $${\Delta G}^{\circ }={\Delta H}^{\circ }-T\cdot {\Delta S}^{\circ }$$

where: ΔG^0^—free Gibbs energy standard variation (kJ mol^−1^), ΔH^0^—standard enthalpy variation (kJ mol^−1^), ΔS^0^—standard entropy variation (J mol^−1^ K^−1^), T—absolute temperature (K).

From linear dependence *ln k_d_* versus *1/T* (depicted in Figure 11) were evaluated the variations of standard enthalpy and entropy:

(3) $$\ln\;K_{d}=\frac{{\Delta S}^{\circ }}{R}-\frac{{\Delta H}^{\circ }}{\mathrm{RT}}$$

where: ΔH^0^—standard enthalpy variation (kJ mol^−1^), ΔS^0^—standard entropy variation (J mol^−1^ K^−1^), T—absolute temperature (K) and R—ideal gas constant (8314 J mol^−1^ K^−1^).

Equilibrium constant was calculated as the ratio between the equilibrium adsorption capacity and the equilibrium concentration:

(4) $$K_{d}=\frac{q_{e}}{C_{e}}$$

Adsorption isotherms represent a useful tool for adsorption process analysis and optimization. Material maximum adsorption capacity was evaluated by modeling obtained experimental data with three different adsorption isotherms: Langmuir isotherm (monolayer adsorption), Freundlich isotherm (developed for heterogeneous surfaces) and Sips isotherm which at limits can describe Langmuir of Freundlich isotherms [51,52,53,54]. Kinetic models are used to identify the adsorption mechanism and to determine speed-limiting stages, including mass transport processes and chemical reactions [55].

Langmuir isotherm is based on three hypotheses: adsorption process is a monolayer adsorption, all superficial active sites are identical, housing one single metallic ion, capacity of one molecule to be adsorbed on surface is independent of the occupancy of the neighboring places [52,56].

Nonlinear form of Langmuir isotherm is expressed by equation [52]:

(5) $$q_{e}=\frac{q_{L}\;K_{L}\;C_{e}}{1+K_{L}\;C_{e}}$$

where: q_e_—equilibrium adsorption capacity (mg g^−1^), C_e_—metallic ion equilibrium concentration (mg L^−1^), q_L_—Langmuir maximum adsorption capacity (mg g^−1^), K_L_—Langmuir constant.

Dimensionless constant R_L_ is characteristic for Langmuir isotherm, being called separation factor or equilibrium parameter, which can be evaluated by using relation:

(6) $$R_{L}=\frac{1}{1+K_{L}\;\mathrm{Co}}$$

where: *R_L_*—separation factor, *K_L_*—Langmuir constant (L mg^−1^), *C_0_*—silver initial concentration (mg L^−1^).

Freundlich isotherm is an empiric one and is used developed for heterogeneous surfaces [51]:

(7) $$q_{e}=K_{F}\;C_{e}^{1/n_{f}}$$

where: *q_e_*—equilibrium adsorption capacity (mg g^−1^), C_e_—metallic ion concentration at equilibrium (mg g^−1^), K_F_ and *n_F_*—characteristic constants, which can be associated with relative adsorption capacity of adsorbent and adsorption intensity, respectively.

Sips model [53], which at its limits describes the Langmuir and Freundlich models, is described by relation:

(8) $$q_{e}=\frac{q_{s}\;K_{S}\;C_{e}^{1/n_{S}}}{1+K_{S}\;C_{e}^{1/n_{S}}}$$

where: *q_s_*—maximum adsorption capacity (mg g^−1^), *K_s_*—constant linked with material adsorption capacity, *n_s_*—heterogeneity factor.

Starting from Sips isotherm parameters was evaluated a dimensionless parameter—separation factor:

(9) $$R_{S}=\frac{1}{1+K_{S}\;C_{0}^{1/n_{S}}}$$

where: *R_s_*—separation factor, *K_s_*—constant linked with material adsorption capacity, *n_s_*—heterogeneity factor, *C_0_*—metallic ions initial concentration.

Essential characteristics of Sips isotherms are determined by Rs values: if Rs > 1 adsorption is unfavorable, Rs = 1 adsorption process is a linear one, 0 < Rs < 1 adsorption process is a favorable one, if Rs = 0 adsorption is an irreversible one. 

## 3. Results and Discussion

### 3.1. Synthesis of Colloidal Silver

Colloidal silver can be obtained by using chemical, physical, photochemical and biological methods. Chemical synthesis consists of the reduction of silver precursors in the presence of some stabilizing agents. Colloidal silver synthesis by reduction of silver nitrate is a two stage process: in first one small silver particles are formed and in second one the dimension of these particles is increasing. 

#### 3.1.1. Influence of Precursor: Reducing Agent

**Figure 2:**
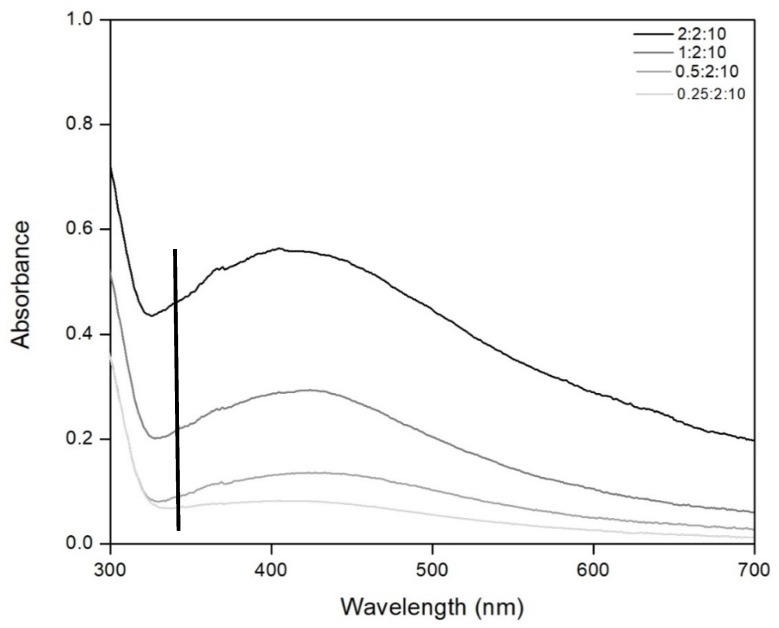
UV–VIS absorption spectra for different precursor:reducing agent: water ratios.

The influence of the ratio of silver precursor:reducing agent during colloidal silver synthesis was studied using 4 different samples with ratios: 0.2:2, 0.5:2, 1:2 and 2:2. After preparation, for all colloidal silver samples the UV–VIS spectra were recorded (Figure 2) in the spectral range of 700–300 nm.

Data presented in Figure 2 show that the recorded UV–VIS spectra presents a large band around 420 nm, which is specific for the presence of colloidal silver particles [2,57].

The reddish brown color became more intense simultaneously with the increase of the ratio of silver precursor:reducing agent, leading at an increase of silver concentration in colloidal solution. The increase of silver concentration is proved by the increase of the specific band from UV–VIS spectra.

Colloidal silver obtained for the ratio of silver precursor:reducing agent being 2:2 has a high viscosity, observing the presence of non-reacted starch. For the ratio of silver precursor:reducing agent being 1:2, it has a normal consistence, without non-reacted starch. Based on this observation, we can conclude that the best results are obtained by using the ratio of silver precursor:reducing agent being 1:2. For any further experiments this ratio is used.

#### 3.1.2. Influence of Ultrasound Time

**Figure 3:**
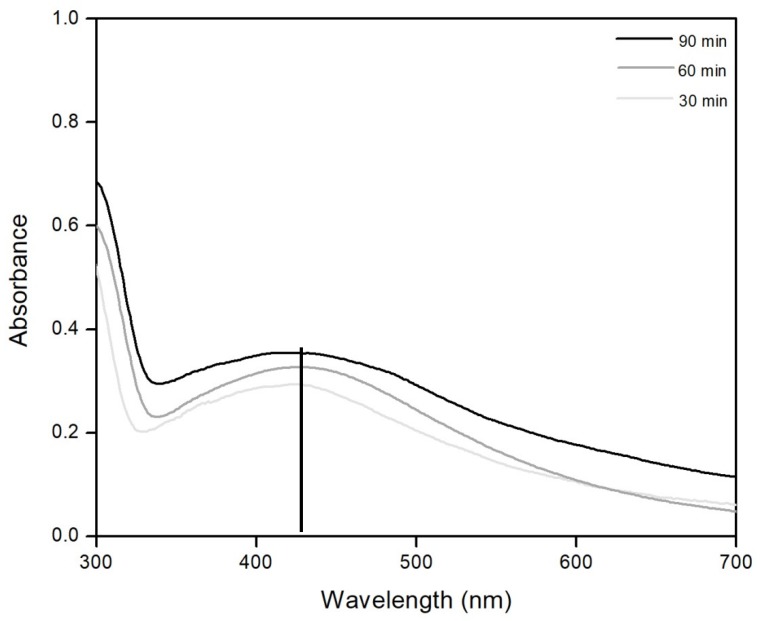
UV–VIS absorption spectra for different ultrasound times.

The optimum sonication time for the synthesis of colloidal silver obtained for the chosen ratio was established (Figure 3). 

Data presented in Figure 3 show the presence of a colloidal silver specific band located at approximately 420 nm for all three used times [2,57]. It can also be observed that the best results are obtained for synthesis conducted for 60 minutes. Based on this observation we decided that all further syntheses will be carried out for 60 minutes.

#### 3.1.3. Influence of Ultrasound Baths Temperature

**Figure 4:**
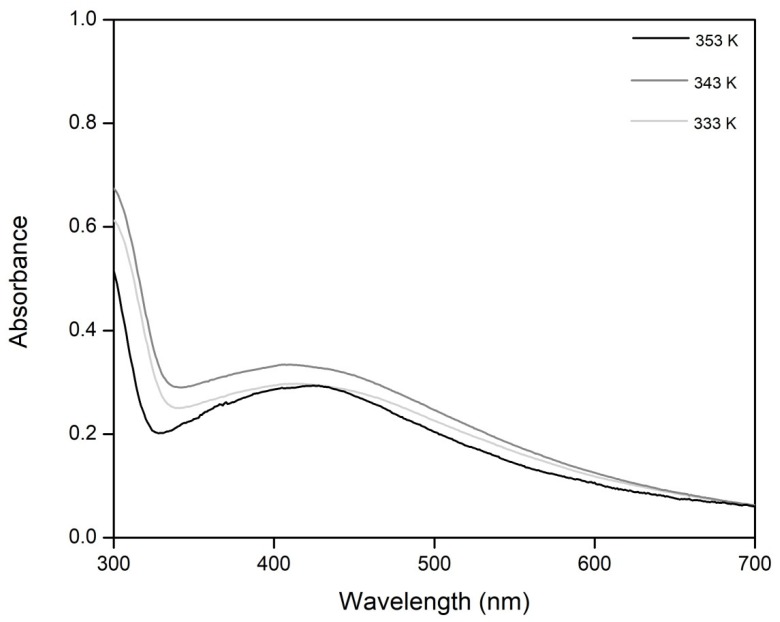
UV–VIS absorption spectra for different ultrasound bath temperatures.

Another important parameter for colloidal silver preparation is synthesis temperature. In order to determine the optimum synthesis temperature, all syntheses were carried out at three different temperatures: 333, 343, 353 K. After preparation, we recorded the VIS spectra in order to prove the presence of silver particles into the prepared colloidal solutions (Figure 4).

From the recorded VIS spectra, it was observed that the increase of temperature has a beneficial effect, leading to an increase of silver concentration in colloidal silver. By corroborating all these results with the recorded SEM micrographs, we can conclude that the optimum temperature for colloidal silver preparation is 353 K, because at this temperature silver particles with low dimensions and relatively good dispersion were obtained.

### 3.2. Characterization of Colloidal Silver Materials

#### SEM and EDX Characterization

**Figure 5:**
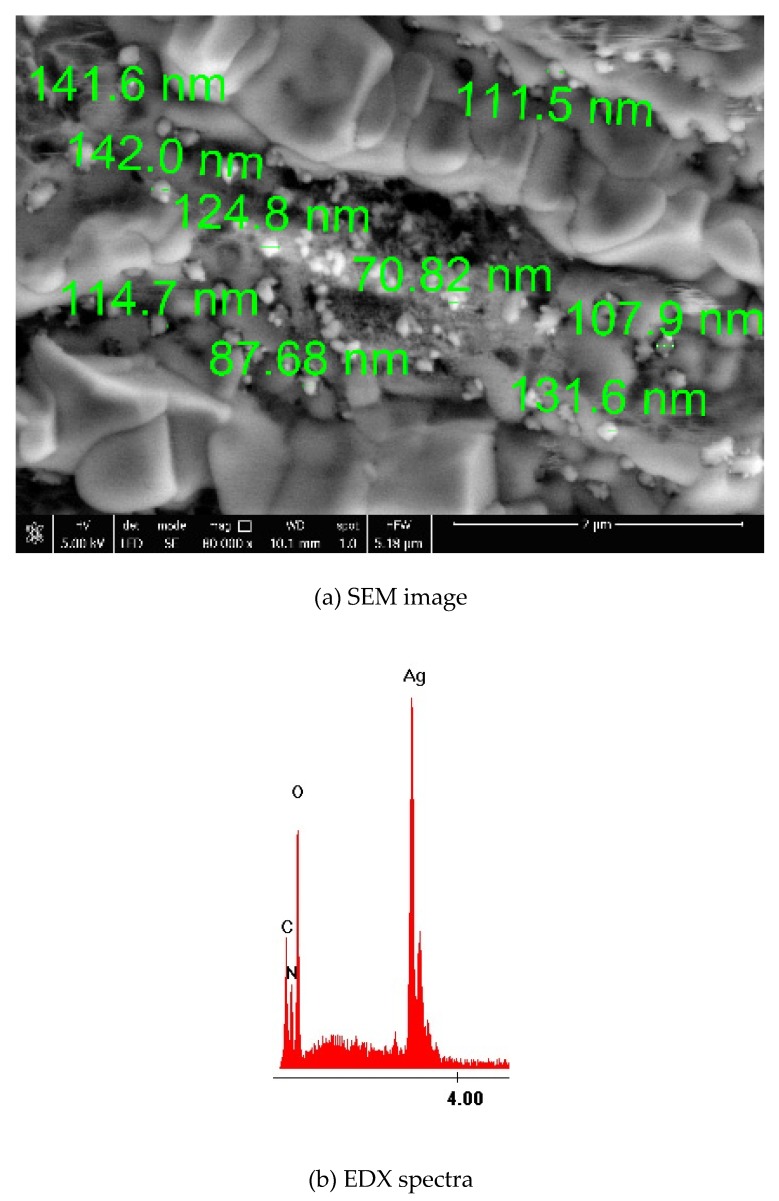
SEM image (**a**) and Energy Dispersive X-ray (EDX) spectra (**b**) for synthetized colloidal silver.

The prepared material was characterized using Scanning Electron Microscopy (SEM) and by Energy Dispersive X-ray spectroscopy (EDX). SEM microscopy was used to analyze surface morphology (Figure 5a) and EDX (Figure 5b) was used to prove the presence of silver ions in the synthesized material.

From the SEM image presented in Figure 5a, the presence of some white spots were observed which were associated with silver presence. Analyzing the SEM picture from Figure 5 we can observe that the silver particles present a size distribution between 70.82 nm and 142.00 nm. Presence of silver particles was proved by recording the EDX spectra, in which the peaks for silver and for starch specific elements were present. Nitrogen presence is associated with incomplete reduction of silver nitrate, or due to its excess. The presence of oxygen atoms in the EDX spectra can be explained by considering that the silver adsorption onto the cotton surface has been realized in air, so during the process some silver atoms get oxidized. 

### 3.3. Application of the Colloidal Silver

#### 3.3.1. Biocide Effect of Colloidal Silver Solution

**Figure 6:**
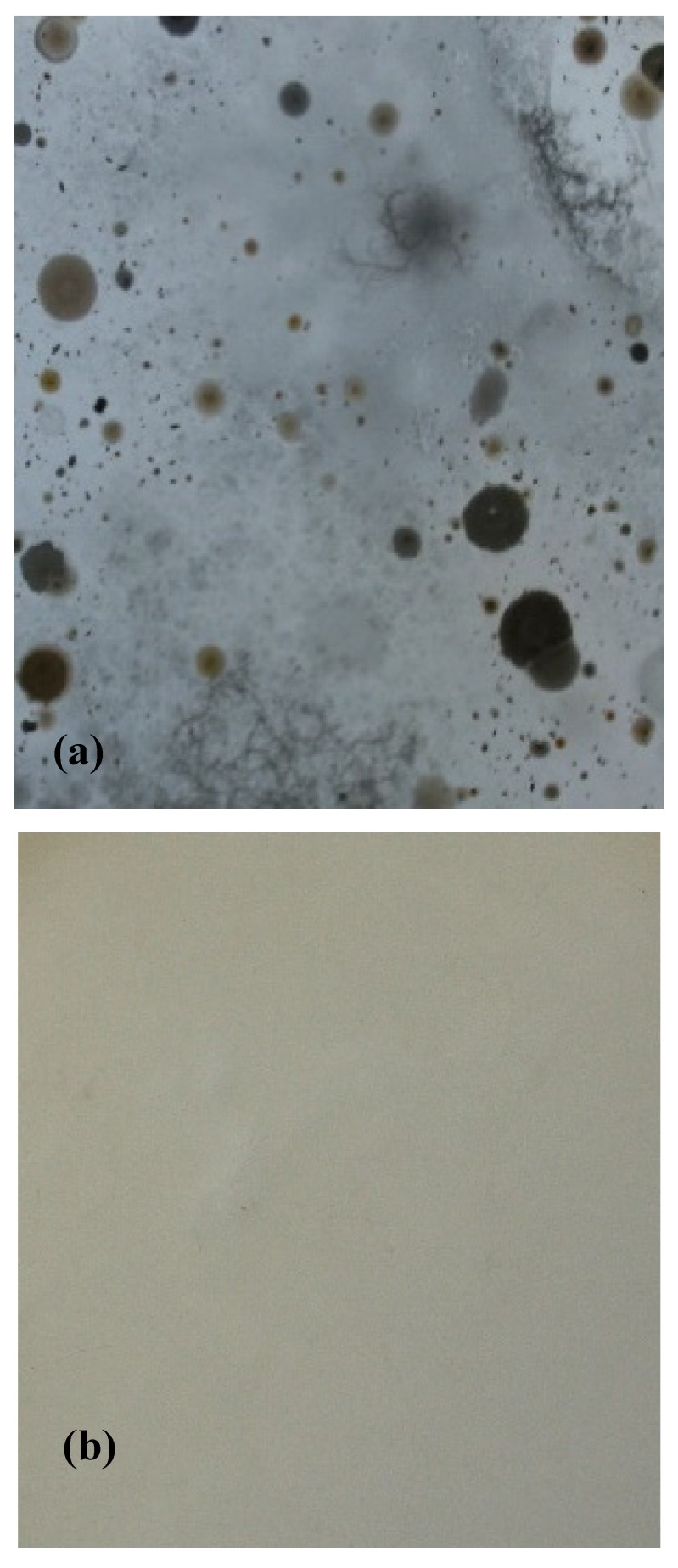

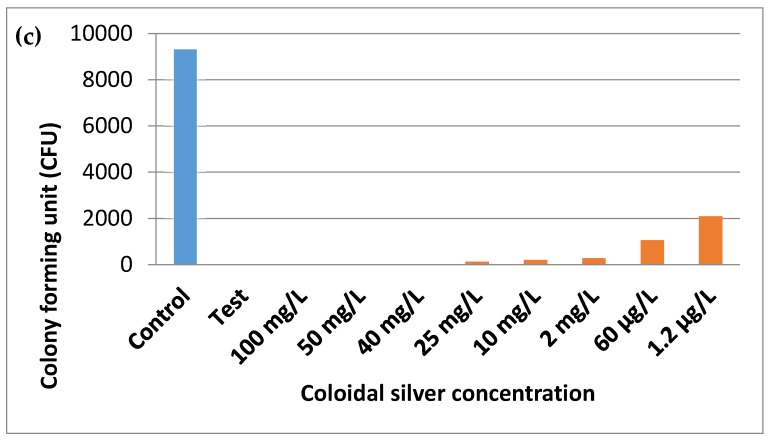
Bacterial inhibition zone image and total colony units. (**a**)—Control; (**b**) —50 mg Ag colloidal/L; (**c**)—CFU vs colloidal silver concentration.

Biocide character of synthesized colloidal silver was performed on a bacterial community obtained from the environment, presenting a large biodiversity and higher resistance to environmental factors. Figure 6 shows the bacterial growth in the control and test cultures. In the control culture (without colloidal silver), the average number of bacterial cells (colony forming unit) per mL was 9320 CFU. In the test sample (with colloidal silver) it was observed that the bacteria grew up when colloidal silver concentration was under 50 mg L^−1^. Based on this observation it was considered that the toxic concentration is 50 mg L^−1^. Due to this, all further experiments were carried out at this silver concentration.

#### 3.3.2. Antibacterial Activities

To prove the antibacterial properties of medical bandages with silver, colloidal silver was deposited on medical bandages by adsorption. For the adsorption process we established specific parameters: ratio solid adsorbent:colloidal solution, contact time, temperature, initial concentration of colloidal silver and the influence of these parameters on adsorption capacity of medical bandages. In addition, the adsorption mechanism by equilibrium, kinetic and thermodynamic studies was established.

#### 3.3.3. Influence of Solid:Liquid Ratio

**Figure 7:**
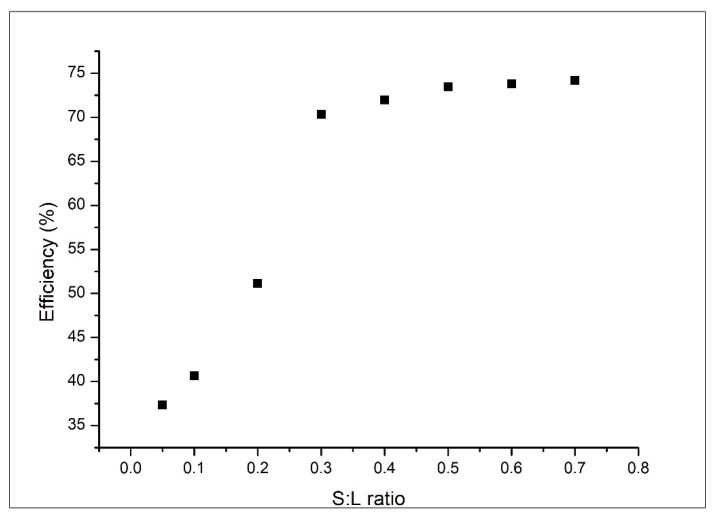
Influence of solid (S):liquid (L) ratio on adsorption process efficiency.

Influence of ratio solid adsorbent:colloidal silver on adsorption capacity was established by changing the numbers of medical bandages immersed into the colloidal silver solution. In these experiments we changed the number of samples introduced in 25 mL of colloidal silver solution with a concentration of 50 mg L^−1^. Experiments were carried out at 298 K, for a contact time of 4 h and pH 4. In Figure 7 is presented the influence of the ratio of solid:liquid over adsorption capacity. 

From Figure 7 we can observe that with the increase of the solid:liquid ratio, adsorption efficiency has increased, until a ratio of solid:liquid = 0.3 g:25 mL when a plateau is reached. This plateau corresponds to an adsorption capacity of 70 %. This ratio corresponds to 30 pieces 20 × 20 mm of medical bandages. Based on this observation all further experiments were carried out by using the optimum solid:liquid ratio.

#### 3.3.4. Influence of Contact Time and Temperature 

**Figure 8:**
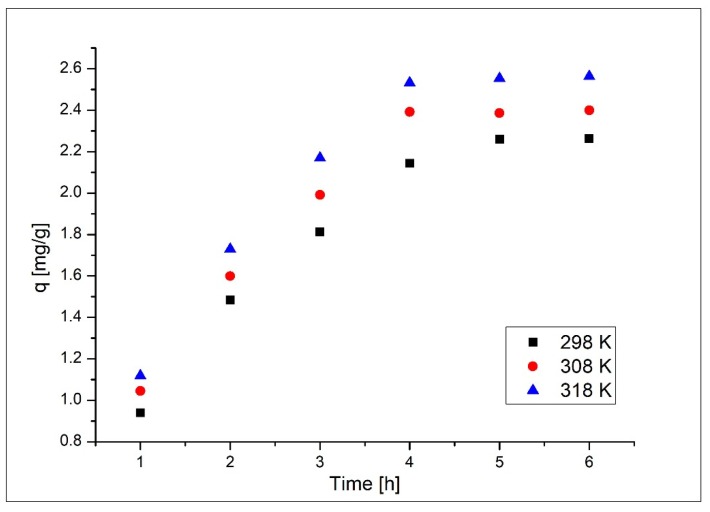
Influence of contact time and temperature on adsorption capacity.

In Figure 8 the influences of contact time and temperature on maximum adsorption capacity are presented. Data presented in Figure 8 reveal that the temperature and time increase present a similar influence on maximum adsorption capacity. When the contact time and temperature increase, the maximum adsorption capacity increases. After 4 h maximum adsorption capacity remains constant. Temperature influence on maximum adsorption capacity does not have a significant influence, so it is not necessarily to work at temperatures higher than 298 K. Based on these observations we chose the optimum conditions: time—4 h and temperature of 298 K.

#### 3.3.5. Kinetics Studies

Based on the obtained experimental data we established the kinetic mechanism for silver adsorption process. Colloidal silver adsorption process kinetics on medical bandages were established by using two kinetics equations: pseudo-first-order model [48] and pseudo-second-order model [49,50]. 

**Figure 9:**
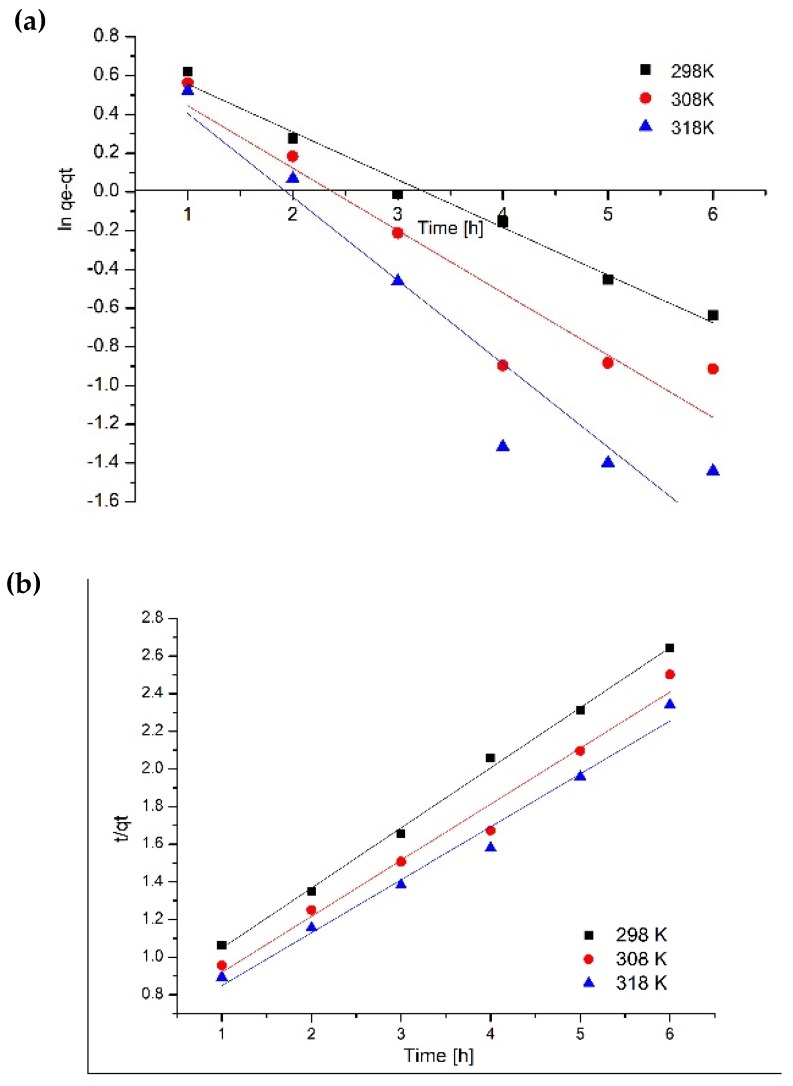
Kinetic studies. (**a**) pseudo-first-order; (**b**) pseudo-second-order.

Experimental data were modeled using the linear forms of these equations. Speed constant for pseudo-first-order model was determined from linear representation of *ln(q_e_ – q_t_)* versus *time*, and the speed constant for pseudo-second-order was determined from linear representation of *t/q_t_* versus *time*. Based on the modeled data presented in Figure 9 we determined the kinetic parameters associated with the adsorption process for used kinetic models (Table 1). Based on the obtained correlation factors we established the kinetic model which had better described the studied adsorption process. 

Analyzing the kinetic parameters obtained when experimental data were modeled with pseudo-first-order model, it was observed that the correlation coefficient is lower than 1, meaning that this kinetic model is not describing the studied adsorption process. In addition, we calculated the maximum adsorption, which had lower values compared with the experimentally obtained ones.

Similarly, the experimental data were modeled with the pseudo-second-order equation in order to prove that the model is describing the colloidal silver adsorption on medical bandages. Kinetic parameters (Table 1) associated with pseudo-second-order were determined from linear dependence of *t/q_t_* versus *time* (Figure 9). From the values of the correlation coefficient close to the unity, it was proved that the pseudo-second-order model is describing the studied adsorption process. Another confirmation is represented by the values of calculated adsorption capacity which are very close to the experimental ones. 

**Figure 10:**
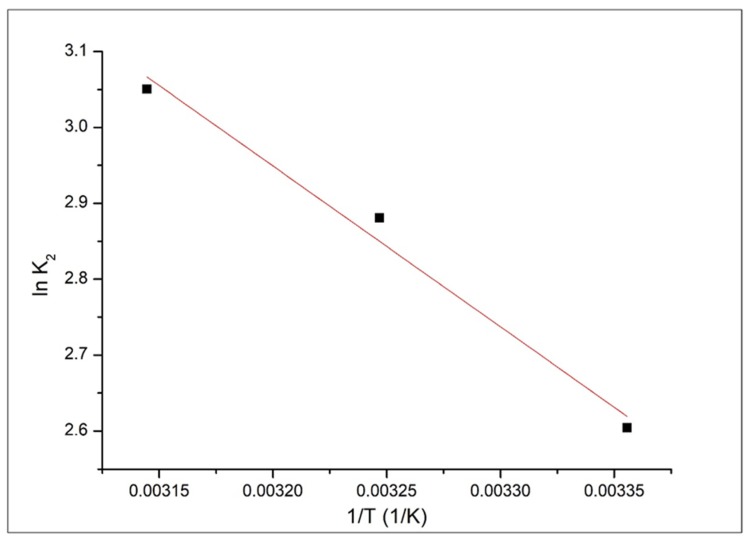
*ln K_2_* = f (*1/T*).

For the studied adsorption process, the activation energy was evaluated from linear dependence of *lnk2* versus *1/T* (Figure 10). In this case, the activation energy was evaluated by using the speed constant obtained for the pseudo-second-order model, which is accurately describing the adsorption process.

Based on the data from Figure 10 we calculated the activation energy for silver adsorption on medical bandages (17.72 kJ mol^−1^). 

#### 3.3.6. Thermodynamics Studies of the Adsorption Process

Thermodynamic studies were carried out in the temperature range of 298 to 318 K in order to confirm if the adsorption process is a spontaneous one. Values of thermodynamic functions are presented in Table 2. 

**Figure 11:**
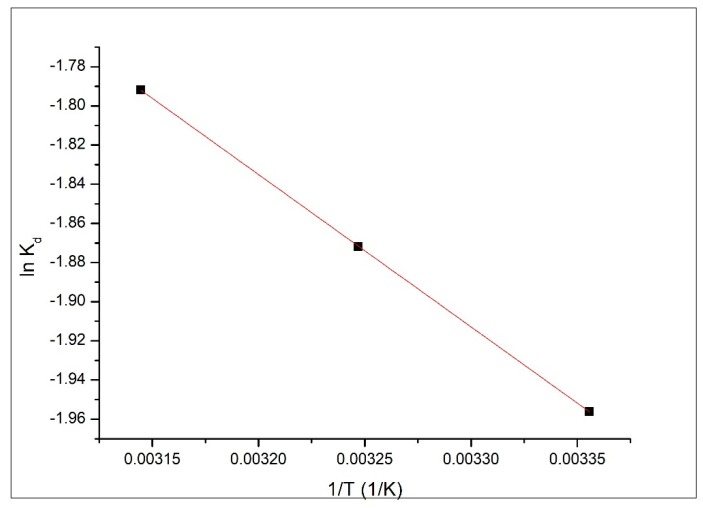
*ln k_d_* = f (*1/T*).

From the linear dependence *ln k_d_* versus *1/T* we evaluated the variations of standard enthalpy and entropy (Figure 11).

Analyzing the data presented in Table 2, we can observe that the Gibbs free energy variation has negative values, meaning that the studied adsorption process is a spontaneous one. Temperature increase leads to a decrease of Gibbs free energy value, confirming that the studied adsorption process is favored by temperature increase. By correlating the small increase of maximum adsorption capacity with temperature increase and positive values of enthalpy, we can conclude that the studied adsorption processes are endothermic. A positive value of entropy variation suggests that the studied adsorption process presents a relatively high disorder at the solid/liquid interface, but the low value of entropy suggests that major changes of disorder degree are not taking place at the liquid/solid interface.

#### 3.3.7. Equilibrium Studies

**Figure 12:**
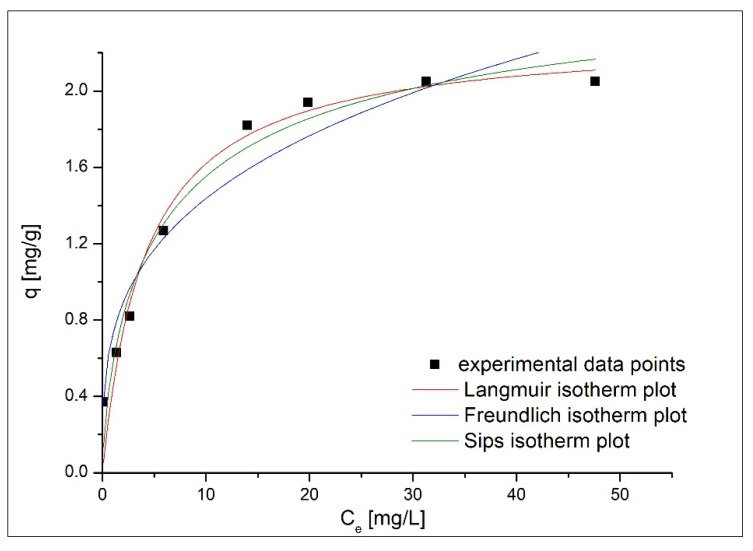
Experimental data modeled with adsorption isotherms.

Figure 12 depicts experimental data modeled with the chosen kinetic isotherms. The obtained kinetic parameters are presented in Table 3.

Based on the data listed in Table 3, it was observed that at higher equilibrium concentrations the medical bandage’s maximum adsorption capacity tends to be a constant value, which is the experimental maximum adsorption capacity (q_exp_—2.35 mg g^−1^).

The parameter 1/n_F_ has a subunit value (1/n_F_—0.29), therefore the used adsorbent presents a good affinity for colloidal silver. Considering the heterogeneity factor 1ns=0.3, which represents a high deviation from unity, we conclude that the medical bandage adsorbent presents a heterogeneous surface. In addition, from the presented data it can observed that the lowest value of the correlation coefficient was obtained when the experimental data were modeled using Freundlich isotherm. In addition, it was observed that the adsorption process is better described by Langmuir isotherm (R^2^ = 0.95616). When experimental data were modeled using the Langmuir isotherm, the calculated maximum adsorption capacity ($q_{L}=2.30\;\mathrm{mg}\;g^{-1}$) is close to the experimentally obtained one ($q_{m,\;exp}=2.35\;\mathrm{mg}\;g^{-1}$), suggesting that the Langmuir isotherm is describing the adsorption process.

#### 3.3.8. The Material Characterization after Silver Colloidal Adsorption 

**Figure 13:**
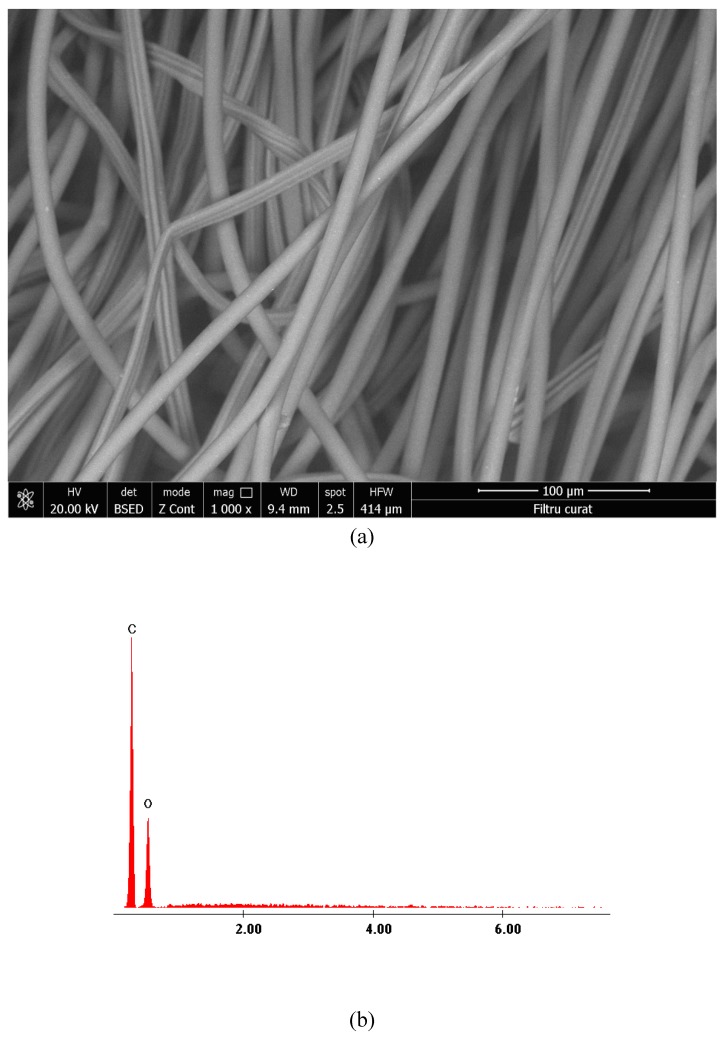

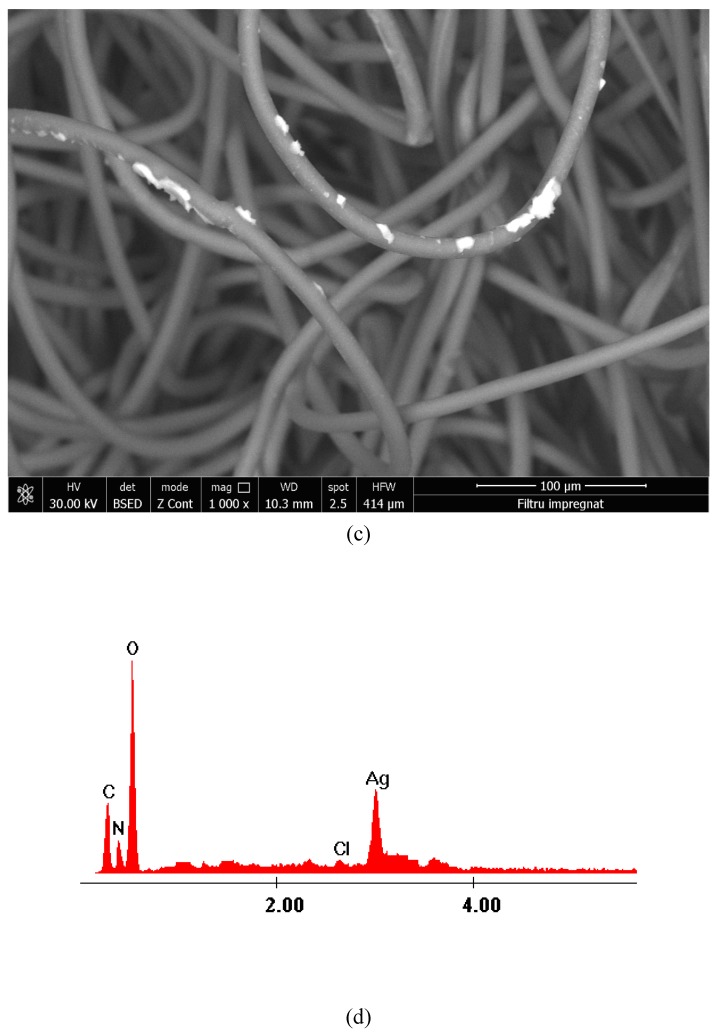
Characterization of the cotton before and after colloidal silver adsorption. (**a**) SEM image before adsorption; (**b**) EDX spectra before adsorption; (**c**) SEM image after adsorption; (**d**) EDX spectra after adsorption.

In order to confirm the presence of silver particles adsorbed onto the cotton medical bandages, they were characterized by using SEM and EDX (micrographs and EDX spectra are presented in Figure 13a–d).

From the recorded SEM images, the presence of silver atoms after adsorption can be observed (Figure 13c), which was also confirmed by EDX spectra because of the silver peak.

#### 3.3.9. Adsorption Process Mechanism—Schematically the Mechanism of Colloidal Silver Adsorption Process is Presented in Figure 14

**Figure 15:**
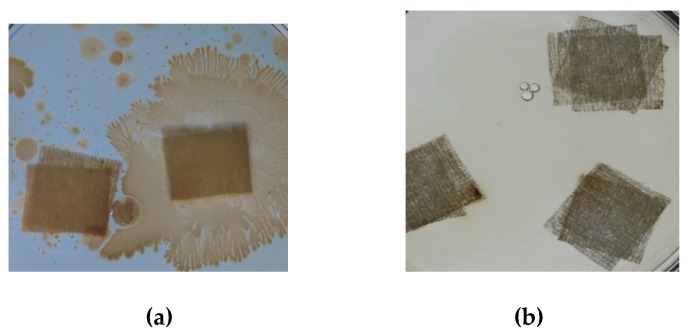
Antimicrobial test. (**a**) Control (**b**) Test.

After we established the optimum conditions for silver adsorption on medical bandages, we tested the antibacterial effect in order to prove its usage in the medical field. In this case material pieces were dried at 303 K for 24 h and used in antibacterial tests (Figure 15). 

For antibacterial tests, pieces of the produced material were placed in Petri dishes at 2 cm from the edges, and were inoculated using 600 μL of inoculum that contains heterotrophs bacteria from the Bega river. After that, in each Petri dish was added sterile plate count agar growth medium. Prepared Petri dishes were incubated at 303 K for 48 hours. After incubation we observed a good bacterial growth for non-impregnated medical bandages (Figure 15 a). Bacterial growth was inhibited when the silver concentration was higher than the minimum toxic concentration.

## 4. Conclusions

In the present paper was proposed a new route for colloidal silver synthesis by ultrasonication, using soluble starch as reducing agent and stabilizer. In this case it was not needed to use a pH regulator. Synthesized colloidal silver was characterized by UV–VIS, SEM and EDX. We investigated adsorption mechanism, and the possible applications of deposited silver particles on medical bandages. The adsorption mechanism was investigated by kinetic, thermodynamic and equilibrium studies. Based on obtained experimental data we determined the optimum adsorption conditions: pH—4, time = 4 h, temperature 298 K. Silver adsorption process is described by Langmuir, when the maximum adsorption capacity was 2.30 mg g^−1^ it was very close to the experimental one. From thermodynamic studies, it was proven that the silver adsorption on medical bandages is an endothermic and spontaneous process. As a possible application, we evaluated the biocide effect of colloidal silver and medical bandages with silver content using heterotrophs bacteria. When the silver concentration was higher than 50 mg L^−1^, it became toxic for heterotrophic bacteria. Similar for medical bandages with silver content it was proven that the toxic concentration for heterotrophs bacteria was 50 mg of silver. 

## Figures and Tables

**Figure 14:**
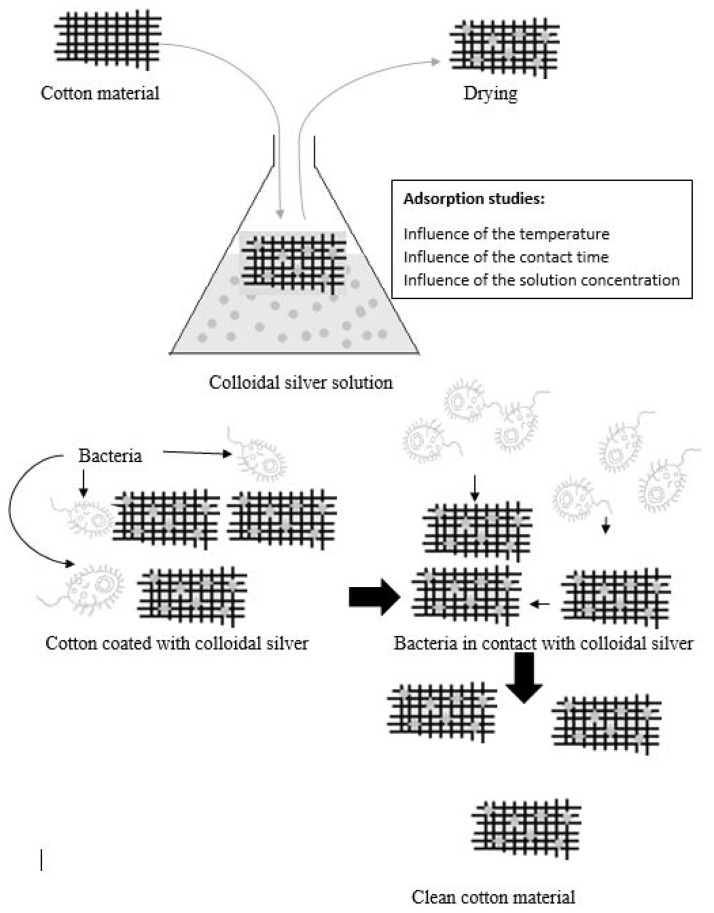
Mechanism of colloidal silver adsorption process.

**Table 1:** Kinetic parameters for the adsorption of colloidal silver onto material/textile/cotton.

**Pseudo-First Order**				
**Temperature (K)**	***q*_e,exp_** **(mg g^−1^)**	***k*_1_** **(min^−1^)**	***q*_e,calc_** **(mg g^−1^)**	**R^2^**
298	2.16	0.246	1.06	0.9839
308	2.38	0.322	1.81	0.8906
318	2.55	0.430	2.03	0.9133
**Pseudo-Second Order**				
**Temperature (K)**	***q*_e,exp_** **(µg g^−1^)**	***k*_2_** **(g mg^−1^∙min^−1^)**	***q*_e,calc_** **(mg g^−1^)**	**R^2^**
298	2.16	13.52	2.58	0.9974
308	2.38	17.83	3.03	0.9803
318	2.55	21.13	3.15	0.9831

**Table 2:** Thermodynamic parameters for adsorption of silver onto cotton.

Δ*H*^0^ (kJ/mole)	Δ*S^0^* (J/mole∙K)	Δ*G^0^* (kJ/mole)			R^2^
5.7	19.24	298 K	308 K	318 K	0.9999
		−11.8	−20.36	−39.68	

**Table 3:** Kinetic parameters obtained for the used adsorption isotherms.

**Langmuir Isotherm**			
***q*_m,exp_ (mg/g)**	***K*_L_ (L/mg)**	***q*_L_ (mg/g)**	***R*^2^**
2.35	0.239	2.30	0.95616
**Freundlich Isotherm**			
***K*_F_ (mg/g)**	**1/*n*_F_**		***R*^2^**
0.725	0.29		0.93262
**Sips Isotherm**			
***K*_S_**	***q*_S_ (mg/g)**	**1/*n*_S_**	***R*^2^**
0.268	2.74	0.3	0.95548

## References

[1] Thamilselvi V., Radha K. (2017). A Review On The Diverse Application Of Silver Nanoparticle. IOSR J. Pharm..

[2] Hiragond C.B., Kshirsagar A.S., Dhapte V.V., Khanna T., Joshi P., More P.V. (2018). Enhanced anti-microbial response of commercial face mask using colloidal silver nanoparticles. Vacuum.

[3] Tran Q.H., Nguyen V.Q., Le A.-T. (2013). Silver nanoparticles: Synthesis, properties, toxicology, applications and perspectives. Adv. Nat. Sci. Nanosci. Nanotechnol..

[4] Marambio-Jones C., Hoek E.M.V. (2010). A review of the antibacterial effects of silver nanomaterials and potential implications for human health and the environment. J. Nanopart. Res..

[5] Enoch D.A., Ludlam H.A., Brown N.M. (2006). Invasive fungal infections: A review of epidemiology and management options. J. Med. Microbiol..

[6] Coker R.J., Hunter B.M., Rudge J.W., Liverani M., Hanvoravongchai P. (2011). Emerging infectious diseases in southeast Asia: Regional challenges to control. Lancet.

[7] Yoon K.Y., Byeon J.H., Park C.W., Hwang J. (2008). Antimicrobial Effect of Silver Particles on Bacterial Contamination of Activated Carbon Fibers. Environ. Sci. Technol..

[8] Sheng Z., Liu Y. (2011). Effects of silver nanoparticles on wastewater biofilms. Water Res..

[9] Yetisen A.K., Qu H., Manbachi A., Butt H., Dokmeci M.R., Hinestroza J.P., Skorobogatiy M., Khademhosseini A., Yun S.H. (2016). Nanotechnology in Textiles. ACS Nano.

[10] Kumar M., Sandeep C.S., Kumar G., Mishra Y.K., Philip R., Reddy G.B. (2014). Plasmonic and Nonlinear Optical Absorption Properties of Ag:ZrO_2_ Nanocomposite Thin Films. Plasmonics.

[11] Cherenack K., Pieterson L.V. (2012). Smart textiles: Challenges and opportunities. J. Appl. Phys..

[12] Anand S.C., Horrocks A.R., Anand S.C. (2016). Technical Fabric Structures—2. Knitted Fabrics. Handbook of Technical Textiles.

[13] Qian L., Sun G. (2003). Durable and regenerable antimicrobial textiles: Synthesis and applications of 3-methylol-2,2,5,5-tetramethyl-imidazolidin-4-one (MTMIO). J. Appl. Polym. Sci..

[14] Paszkiewicz M., Gołąbiewska A., Rajski Ł., Kowal E., Sajdak A., Zaleska-Medynska A. (2016). The Antibacterial and Antifungal Textile Properties Functionalized by Bimetallic Nanoparticles of Ag/Cu with Different Structures. J. Nanomater..

[15] Gupta P., Bajpai M., Bajpai S. (2008). Textile technology: Investigation of antibacterial properties of silver nanoparticle-loaded poly (acrylamide-co-itaconic acid)-grafted cotton fabric. J. Cotton Sci..

[16] Juknius T., Ružauskas M., Tamulevičius T., Šiugždinienė R., Juknienė I., Vasiliauskas A., Jurkeviit A., Tamuleviius S. (2016). Antimicrobial Properties of Diamond-Like Carbon/Silver Nanocomposite Thin Films Deposited on Textiles: Towards Smart Bandages. Materials.

[17] Xiao A., Dhand C., Leung C.M., Beuerman R.W., Ramakrishna S., Lakshminarayanan R. (2018). Strategies to design antimicrobial contact lenses and contact lens cases. J. Mater. Chem. B.

[18] Zakarienė G., Novoslavskij A., Meškinis Š., Vasiliauskas A., Tamulevičienė A., Tamulevičius S., Alter T., Malakauskas M. (2018). Diamond like carbon Ag nanocomposites as a control measure against Campylobacter jejuni and Listeria monocytogenes on food preparation surfaces. Diam. Relat. Mater..

[19] Ramyadevi J., Jeyasubramanian K., Marikani A., Rajakumar G., Rahuman A.A. (2012). Synthesis and antimicrobial activity of copper nanoparticles. Mater. Lett..

[20] Ren G., Hu D., Cheng E.W., Vargas-Reus M.A., Reip P., Allaker R.P. (2009). Characterisation of copper oxide nanoparticles for antimicrobial applications. Int. J. Antimicrob. Agents.

[21] Mishra Y.K., Adelung R. (2018). ZnO tetrapod materials for functional applications. Mater. Today.

[22] Stanić V., Dimitrijević S., Antić-Stanković J., Mitrić M., Jokić B., Plećaš I.B., Raičević S. (2010). Synthesis, characterization and antimicrobial activity of copper and zinc-doped hydroxyapatite nanopowders. Appl. Surf. Sci..

[23] Schabes-Retchkiman P.S., Canizal G., Herrera-Becerra R., Zorrilla C., Liu H.B., Ascencio J.A. (2006). Biosynthesis and characterization of Ti/Ni bimetallic nanoparticles. Opt. Mater..

[24] Martinez-Gutierrez F., Olive P.L., Banuelos A., Orrantia E., Nino N., Sanchez E.M., Ruiz F., Bach H., Av-Gay Y. (2010). Synthesis, characterization, and evaluation of antimicrobial and cytotoxic effect of silver and titanium nanoparticles. Nanomed. Nanotechnol. Biol. Med..

[25] Lellouche J., Friedman A., Lahmi R., Gedanken A., Banin E. (2012). Antibiofilm surface functionalization of catheters by magnesium fluoride nanoparticles. Int. J. Nanomed..

[26] Perni S., Piccirillo C., Pratten J., Prokopovich P., Chrzanowski W., Parkin I.P., Wilson M. (2009). The antimicrobial properties of light-activated polymers containing methylene blue and gold nanoparticles. Biomaterials.

[27] Qi L., Xu Z., Jiang X., Hu C., Zou X. (2004). Preparation and antibacterial activity of chitosan nanoparticles. Carbohydr. Res..

[28] Singh R., Lillard J.W. (2009). Nanoparticle-based targeted drug delivery. Exp. Mol. Pathol..

[29] Wahab R., Dwivedi S., Khan F., Mishra Y.K., Hwang I.H., Shin H.S., Musarrat J., Al-Khedhairy A.A. (2014). Statistical analysis of gold nanoparticle-induced oxidative stress and apoptosis in myoblast (C2C12) cells. Coll. Surf. B Biointerfaces.

[30] Wiley B.J., Im S.H., Li Z.Y., McLellan J., Siekkinen A., Xia Y. (2006). Maneuvering the Surface Plasmon Resonance of Silver Nanostructures through Shape-Controlled Synthesis. J. Phys. Chem. B.

[31] Iravani S., Korbekandi H., Mirmohammadi S.V., Zolfaghari B. (2014). Synthesis of silver nanoparticles: Chemical, physical and biological methods. Res. Pharm. Sci..

[32] Tarasenko N.V., Butsen A.V., Nevar E.A., Savastenko N.A. (2006). Synthesis of nanosized particles during laser ablation of gold in water. Appl. Surf. Sci..

[33] Kawasaki M., Nishimura N. (2006). 1064-nm laser fragmentation of thin Au and Ag flakes in acetone for highly productive pathway to sTable metal nanoparticles. Appl. Surf. Sci..

[34] Hussain J.I., Kumar S., Hashmi A.A., Khan Z. (2011). Silver nanoparticles: Preparation, characterization, and kinetics. Adv. Mater. Lett..

[35] Al-Mubaddel F.S., Haider S., Al-Masry W.A., Al-Zeghayer Y., Imran M., Haider A., Ullah Z. (2017). Engineered nanostructures: A review of their synthesis, characterization and toxic hazard considerations. Arab. J. Chem..

[36] Sato-Berrú R., Redón R., Vázquez-Olmos A., Saniger J.M. (2009). Silver nanoparticles synthesized by direct photoreduction of metal salts. Application in surface-enhanced Raman spectroscopy. J. Raman Spectrosc..

[37] Ghosh S.K., Kundu S., Mandal M., Nath S., Pal T. (2003). Studies on the Evolution of Silver Nanoparticles in Micelle by UV-Photoactivation. J. Nanopart. Res..

[38] Huang L., Zhai M.L., Long D.W., Peng J., Xu L., Wu G.Z., Li J.Q., Wei G.S. (2008). UV-induced synthesis, characterization and formation mechanism of silver nanoparticles in alkalic carboxymethylated chitosan solution. J. Nanopart. Res..

[39] Veerasamy R., Xin T.Z., Gunasagaran S., Xiang TF W., Yang EF C., Jeyakumar N., Dhanaraj S.A. (2011). Biosynthesis of silver nanoparticles using mangosteen leaf extract and evaluation of their antimicrobial activities. J. Saudi Chem. Soc..

[40] Lee S.H., Jun B.-H. (2019). Silver Nanoparticles: Synthesis and Application for Nanomedicine. Int. J. Mol. Sci..

[41] Pantidos N., Horsfall L. (2014). Biological Synthesis of Metallic Nanoparticles by Bacteria, Fungi and Plants. J. Nanomed. Nanotechnol..

[42] Firdhouse J., Lalitha P. (2015). Biosynthesis of Silver Nanoparticles and Its Applications. J. Nanotechnol..

[43] Prabhu S., Poulose E.K. (2012). Silver nanoparticles: Mechanism of antimicrobial action, synthesis, medical applications, and toxicity effects. Int. Nano Lett..

[44] Nasrollahzadeh M. (2014). Green synthesis and catalytic properties of palladium nanoparticles for the direct reductive amination of aldehydes and hydrogenation of unsaturated ketones. New J. Chem..

[45] Ju L., Wan Y., Wang X., Liang Q., Li Z., Xu S. (2016). Efficient visible light photocatalytic activitiy of tetranitro substituted cobalt phthalocyanines–attapulgite hybrid materials fabricated by ultrasonic impregnation method. Optik.

[46] Barathi M., Kumar A.S.K., Rajesh N. (2014). A novel ultrasonication method in the preparation of zirconium impregnated cellulose for effective fluoride adsorption. Ultrason. Sonochem..

[47] Sawant S.S., Anil A.C., Krishnamurthy V., Gaonkar C., Kolwalkar J., Khandeparker L., Desai D., Mahulkar A.V., Ranade V.V., Pandit A.B. (2008). Effect of hydrodynamic cavitation on zooplankton: A tool for disinfection. Biochem. Eng. J..

[48] Lagergren S. (1898). About the theory of so-called adsorption of soluble substabces. Sven. Vetenskapsakad. Handingarl.

[49] Ho Y.S., McKay G. (1998). A Comparison of Chemisorption Kinetic Models Applied to Pollutant Removal on Various Sorbents. Process Saf. Environ. Prot..

[50] Ho Y.S., Mckay G. (1998). The kinetics of sorption of basic dyes from aqueous solution by sphagnum moss peat. Can. J. Chem. Eng..

[51] Freundlich H.M.F. (1906). Over the adsorption in solution. J. Phys. Chem..

[52] Langmuir I. (1918). The adsorption of gases on plane surfaces of glass, mica and platinum. J. Am. Chem. Soc..

[53] Sips R. (1948). On the Structure of a Catalyst Surface. J. Chem. Phys..

[54] Sert S., Kütahyali C., İnan S., Talip Z., Çetinkaya B., Eral M. (2008). Biosorption of lanthanum and cerium from aqueous solutions by Platanus orientalis leaf powder. Hydrometallurgy.

[55] Bhalara P.D., Punetha D., Balasubramanian K. (2014). A review of potential remediation techniques for uranium(VI) ion retrieval from contaminated aqueous environment. J. Environ. Chem. Eng..

[56] Gabor A., Davidescu C.M., Negrea A., Ciopec M., Grozav I., Negrea P., Duteanu N. (2017). Optimizing the lanthanum adsorption process onto chemically modified biomaterials using factorial and response surface design. J. Environ. Manag..

[57] Rajaboopathi S., Thambidurai S. (2018). Evaluation of UPF and antibacterial activity of cotton fabric coated with colloidal seaweed extract functionalized silver nanoparticles. J. Photochem. Photobiol. B Biol..

